# Development and Validation of a Comprehensive Multivariate Dosimetric Model for Predicting Late Genitourinary Toxicity Following Prostate Cancer Stereotactic Body Radiotherapy

**DOI:** 10.3389/fonc.2020.00786

**Published:** 2020-05-20

**Authors:** Luca F. Valle, Dan Ruan, Audrey Dang, Rebecca G. Levin-Epstein, Ankur P. Patel, Joanne B. Weidhaas, Nicholas G. Nickols, Percy P. Lee, Daniel A. Low, X. Sharon Qi, Christopher R. King, Michael L. Steinberg, Patrick A. Kupelian, Minsong Cao, Amar U. Kishan

**Affiliations:** ^1^Department of Radiation Oncology, University of California, Los Angeles, Los Angeles, CA, United States; ^2^David Geffen School of Medicine, University of California, Los Angeles, Los Angeles, CA, United States

**Keywords:** dose volume histogram (DVH), prostate cancer, multivariate, prediction model, late toxicity, stereotactic body radiation therapy, machine learning

## Abstract

**Purpose:** Dosimetric predictors of toxicity after Stereotactic Body Radiation Therapy (SBRT) are not well-established. We sought to develop a multivariate model that predicts Common Terminology Criteria for Adverse Events (CTCAE) late grade 2 or greater genitourinary (GU) toxicity by interrogating the entire dose-volume histogram (DVH) from a large cohort of prostate cancer patients treated with SBRT on prospective trials.

**Methods:** Three hundred and thirty-nine patients with late CTCAE toxicity data treated with prostate SBRT were identified and analyzed. All patients received 40 Gy in five fractions, every other day, using volumetric modulated arc therapy. For each patient, we examined 910 candidate dosimetric features including maximum dose, volumes of each organ [CTV, organs at risk (OARs)], V100%, and other granular volumetric/dosimetric indices at varying volumetric/dosimetric values from the entire DVH as well as ADT use to model and predict toxicity from SBRT. Training and validation subsets were generated with 90 and 10% of the patients in our cohort, respectively. Predictive accuracy was assessed by calculating the area under the receiver operating curve (AROC). Univariate analysis with student *t*-test was first performed on each candidate DVH feature. We subsequently performed advanced machine-learning multivariate analyses including classification and regression tree (CART), random forest, boosted tree, and multilayer neural network.

**Results:** Median follow-up time was 32.3 months (range 3–98.9 months). Late grade ≥2 GU toxicity occurred in 20.1% of patients in our series. No single dosimetric parameter had an AROC for predicting late grade ≥2 GU toxicity on univariate analysis that exceeded 0.599. Optimized CART modestly improved prediction accuracy, with an AROC of 0.601, whereas other machine learning approaches did not improve upon univariate analyses.

**Conclusions:** CART-based machine learning multivariate analyses drawing from 910 dosimetric features and ADT use modestly improves upon clinical prediction of late GU toxicity alone, yielding an AROC of 0.601. Biologic predictors may enhance predictive models for identifying patients at risk for late toxicity after SBRT.

## Introduction

The recently published HYPO-RT-PC trial provides level I evidence that ultrahypofractionated radiotherapy for prostate cancer offers similar 5-year biochemical control and toxicity rates compared with conventionally fractionated radiotherapy (1). Additional pooled data from phase II trials (2) as well as a large meta-analysis (3) support the specific use of stereotactic body radiotherapy (SBRT), wherein five or fewer fractions of radiotherapy are delivered, generally with the utilization of intensity modulated radiotherapy and image-guided radiotherapy technologies. While the rates of long-term severe toxicity [i.e., grade 3 or greater on the Radiation Therapy Oncology Group (RTOG) or Common Terminology Criteria for Adverse Events (CTCAE) scales] are low, they still occur, and lower grade toxicities—which may not require intervention but can nonetheless degrade quality of life—may occur in a notable minority of patients. These toxicities are likely dose-dependent, and in fact two recently published prospective studies have suggested a toxicity dose-response for prostate SBRT (4, 5). Both studies, along with a large multi-institutional analysis (6), have also implied increased efficacy of prostate cancer ablation with higher doses. As such, doses of up to 40 Gy in five fractions may become increasingly utilized.

Dosimetric predictors of grade 2 or greater toxicity at ablative doses of radiation are not well-established. In fact, most SBRT constraints have been derived based on radiobiological theory (α/β or BED equivalence calculations made after adjustments for longer courses) and *post-hoc* analyses of relatively small cohorts (7). Existing models (8) are limited by low patient numbers, low numbers of events, treatment delivery with an empty bladder, and evaluation of arbitrary dose cut-points rather than evaluation of the entire dose-volume histogram (DVH).

Since 2010, our institution has routinely prescribed 40 Gy using gantry-based SBRT in prospective studies for low- and intermediate-risk disease (NCT01059513) as well as high-risk disease (NCT02296229). As we have consistently used the same planning criteria, treatment delivery techniques, and image guidance protocols, we had the novel opportunity to comprehensively interrogate the entirety DVH in addition to ADT use from a large cohort of prospectively treated patients to identify and validate potential predictors of toxicity.

## Methods and Materials

Three hundred and thirty-nine patients treated with SBRT at our tertiary academic institution from 2010 to 2017 with late CTCAE toxicity data were identified and included in our analysis. All patients were instructed to have a full bladder and empty rectum at the time of computed tomography (CT) simulation with 1.5 mm slice thickness; three fiducial markers were placed transperineally under ultrasound guidance prior to simulation. The clinical target volume (CTV) was the prostate alone and a planning target volume (PTV) was generated by using 5 mm isotropic margins. All patients received 40 Gy in five fractions, every other day, using volumetric modulated arc therapy with four co-planar half-arcs. Ninety-five percent of the PTV was required to receive 40 Gy. A cone beam CT was obtained prior to each fraction to verify stable anatomy, and planar X-ray imaging was obtained before each half-arc with rigid registration to the implanted fiducials. Planning constraints for organs-at-risk have been described previously (9) and the median and interquartile ranges for bladder and rectum dosimetry achieved in the 339 patients are depicted in Table 1. Grade ≥2 late toxicity was assessed according to the GU domain of the CTCAE, version 4.03 and was defined as the worst CTCAE grade scored.

**Table 1:** Median and interquartile range of prostate SBRT planning dosimetry.

**PTV goal**	
	V40 Gy = 95% (95–95%)
**Rectal constraints**	
	V20 Gy = 19.8% (15.6–24.5%)
	V36 Gy = 2.9cc (2.3–3.8cc)
	V40 Gy = 1.1cc (0.6–1.5cc)
**Bladder constraints**	
	V20 Gy = 14.5% (9.3–20.6%)
	V40 Gy = 6.9% (4.3–9.9%)

*cc, cubic centimeters*.

Late grade ≥2 GU toxicity was selected for this analysis due to the high event rate of 20.1% (68/339) in our series (Supplemental Figure 1) compared to toxicity event rates for acute GU toxicity (15/343, 4.4%), acute GI toxicity (7/343, 2.0%), and late GI toxicity (17/339, 5.0%).

We examined 910 candidate dosimetric features including maximum dose (Dmax), V100%, volumes of each organ [CTV, organs at risk (OARs)] and other granular volumetric/dosimetric indices at varying volumetric/dosimetric values from the DVH to differentiate between patients with and without toxicity from SBRT. As ADT has been shown to influence late GU toxicity, this was also included in our model.

For all analyses, 90% of the patients were included in a training subset, whereas the remaining 10% were used in the validation subset. Imbalances between the two groups of patients were addressed by imposing either uniform prior or weighted cost in the toxicity classifier optimization. A 10-fold cross validation was also performed for the purpose of selecting structural hyper-parameters corresponding to model complexity. Once the model structure was fixed, a leave-one-out procedure was used to generate classification scores of each sample in the whole cohort, allowing for subsequent calculation of a receiver operating curve (ROC). In addition to the area under ROC (AROC), we also report the specificity and sensitivity at the given dosimetric threshold, corresponding to the maximum Youden's index. We also visualize the full ROC curve to provide a complete characterization of model accuracy beyond the optimal operating point.

Univariate analysis with student *t*-test was first performed on each candidate DVH feature to identify differences between presence vs. absence of toxicity. The strength of each feature as a stand-alone classifier was also assessed to determine the 10 variables with the highest AROC.

We then used several multivariate analysis methods to assess whether toxicity prediction could be improved, ranging from the most commonly accepted multivariate logistic regression to the more flexible ensemble trees. First, in order to establish baseline multivariate performance, we performed multivariate logistic regression with Least Absolute Shrinkage and Selection Operator (LASSO) regularization. Next, in order to train a toxicity tree for enhanced stability during cross-validation, an optimized classification and regression tree (CART) analysis was performed. Predictor importance was estimated by summing the changes in performance due to optimal splits in the tree.

To further explore methodologies that would improve our predictive capabilities, we then utilized two classes of multivariate ensemble approaches. In the random forest approach, we trained multiple deep decision trees in parallel, each with a randomly drawn (with replacement) subset of covariates and observation from the complete cohort. We optimized the number of trees and random degrees of predictor selection during cross-validation. In order to achieve representation such that high variance was aligned to coordinates where tree decision boundaries lie perpendicular to coordinates of high variance, we added principal component analysis (PCA) preprocessing to the random forest. Original dimensionality was maintained and we allowed the random forest to cope by random sampling of the predictors in each tree component. In the boosted tree approach, we used adaptive boosting to assemble shallow tree learners. Once again, the maximum number of splits and learning rates were optimized during the cross validation process.

Finally, we used a multilayer neural network perception model in attempt to improve accuracy of our toxicity prediction. Two hidden layers were used and the number of nodes in each layer was optimized in the cross validation process to control model complexity.

## Results

Patient characteristics from our cohort are summarized in Table 2. Median follow-up time was 32.3 months (range 3–98.9 months).

**Table 2:** Patient and treatment characteristics.

**Age**	
Mean (standard deviation), years	69.6 (7.6)
Median (range), years	71 (45–92)
**NCCN risk group**	
Low risk	71 (21.0%)
Favorable intermediate risk	107 (31.6%)
Unfavorable intermediate risk	123 (36.3%)
High risk	38 (11.2%)
**Pretreatment PSA**	
Mean (standard deviation), ng/ml	7.7 (4.8)
Median (range), ng/ml	6.6 (0.05–47)
**Gleason grade group**	
I	88 (26.0%)
II	108 (31.9%)
III	107 (31.5%)
IV	22 (6.5%)
V	14 (4.1%)
**T-stage**	
T1c	252 (74.3%)
T2a	69 (20.4%)
T2b	11 (3.2%)
T2c	4 (1.2%)
T3a	2 (0.6%)
T3b	0
T4	0
**Prostate gland size**	
Mean (standard deviation), cc	46.28 (26.9)
Median (range), cc	40 (8.04–263)
**TURP prior to SBRT**	
Yes	11 (3.2%)
**Dose in 5 fractions**	
Mean (standard deviation), Gy	40 (0)
Median (range), Gy	40 (40–40)
**ADT use**	
All patients	43 (12.5%)
Favorable intermediate risk patients	5 (11.6%)
Unfavorable intermediate risk patients	14 (11.2%)
High risk patients	24 (63.2%)
**Duration of ADT with SBRT**	
Mean (standard deviation), months	7.4 (2.5)
Median (range), months	9 (3-12)

*NCCN, National Comprehensive Cancer Network; PSA, Prostate Specific Antigen; TURP, Trans-Urethral Resection of Prostate; SBRT, Stereotactic Body Radiation Therapy; ADT, Androgen Deprivation Therapy*.

Considering the candidate dosimetric features in isolation, the top 10 features for predicting acute grade 2 or greater GU toxicity, ranked by AROC, are presented in Table 3. The top dosimetric features were all related to the rectum, albeit in an inverse fashion, correlating lower rectal dose with higher incidence of late GU toxicity. For example, in the cohort of patients experiencing toxicity, the average rectal V_41.3Gy_ for all patients was 0.303cc whereas the rectal V_41.3Gy_ was 0.43cc in the cohort not experiencing toxicity. However, even the top features identified by univariate analysis poorly discriminated between patients who developed toxicity and patients who did not, with no univariate predictor AROC values exceeding 0.599.

**Table 3:** Top 10 AUCs on univariate analysis.

**Parameter**	**Threshold (cc)**	**Mean cc (SD) in toxicity group**	**Mean cc (SD) in No toxicity group**	***p*-value (*t*-test)**	**Specificity**	**Sensitivity**	**AROC**
Rectum V41.3 Gy	0.205	0.303 (0.372)	0.430 (0.453)	0.034	0.559	0.605	0.599
Rectum V41.4 Gy	0.155	0.263 (0.343)	0.379 (0.422)	0.038	0.544	0.601	0.596
Rectum V41.2 Gy	0.255	0.348 (0.398)	0.483 (0.482)	0.034	0.574	0.609	0.596
Rectum V41.5 Gy	0.125	0.22 (0.314)	0.331 (0.391)	0.042	0.574	0.579	0.594
Rectum V41.7 Gy	0.025	0.164 (0.258)	0.246 (0.327)	0.056	0.471	0.716	0.594
Rectum V41.1 Gy	0.305	0.397 (0.425)	0.539 (0.510)	0.035	0.574	0.620	0.594
Rectum V41.6 Gy	0.045	0.194 (0.286)	0.287 (0.359)	0.049	0.471	0.701	0.593
Rectum V41.9 Gy	0.015	0.113 (0.207)	0.173 (0.260)	0.076	0.529	0.620	0.593
Rectum V0.8 Gy	62.94	67.29 (32.93)	82.48 (126.5)	0.327	0.618	0.594	0.590
Rectum V0.9 Gy	62.75	66.38 (31.58)	81.83 (126.5)	0.319	0.632	0.579	0.590

*SD, Standard deviation; AROC, Area under the receiver operating characteristic curve; cc, cubic centimeters*.

**Figure 1:**
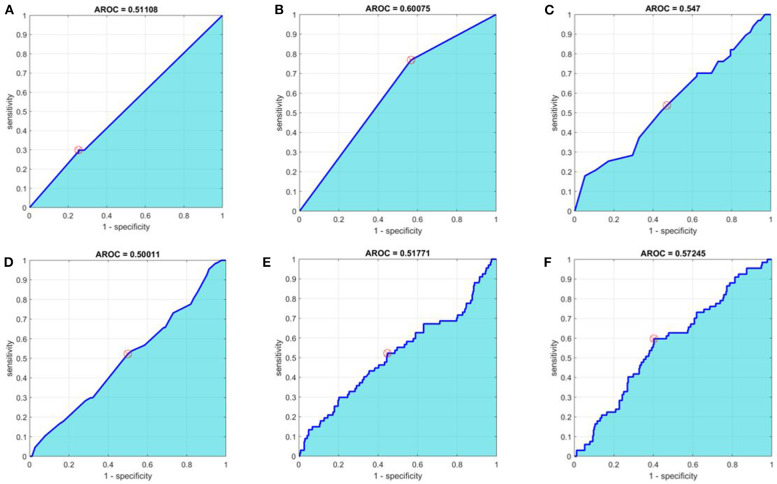
Receiver operating characteristic curves for **(A)** baseline multivariate analysis, **(B)** optimal Classification and Regression Tree (CART), **(C)** Random Forest, **(D)** Principal Component Analysis + Random Forest, **(E)** Boosted Tree, and **(F)** Neural Network methods. Area under the receiver operating characteristic curves (AROC) appears in cyan. The optimal operating point is denoted with a circular red target.

In order to improve on the predictive power of our model, we employed advanced methods, including machine learning, to refine the predictor set with the goal of increasing AROC. The sensitivity, specificity, and AROC for these methods are shown in Table 4. Only the optimized CART model provided a higher AROC than the univariate analyses alone, with an AROC of 0.601 (optimized tree can be found in Supplemental Figure 1). ROC curves for all six advanced methodologies are represented in Figure 1.

**Table 4:** Performance metrics for advanced multivariate prediction methods.

**Method**	**Specificity**	**Sensitivity**	**AROC**
Baseline multivariate analysis	0.746	0.299	0.511
Optimal CART	0.433	0.769	0.601
Random forest	0.530	0.537	0.547
Principal component analysis + random forest	0.500	0.522	0.500
Boosted tree	0.552	0.522	0.518
Multilayer neural network	0.597	0.597	0.572

*AROC, Area under the receiver operating characteristic curve. CART, Classification and Regression Tree*.

## Discussion

We report an attempt at identifying robust dosimetric metrics for predicting grade ≥2 late GU toxicity in a cohort of 339 patients treated with SBRT on prospective trials. To our knowledge, this is the first study to examine candidate features from the entire DVHs of the bladder, rectum, and prostate with a machine learning approach to identify potential dosimetric parameters of importance, with the aim of providing useful information on the dosimetric limitations for mitigating toxicity in patients undergoing SBRT for prostate cancer. We incorporated ADT use in our toxicity prediction model as well. It is highly unique to have 910 individual dosimetric features available for analysis, and even in this context, our study did not identify any high-performing predictors after considering singular DVH parameters or ADT use in isolation. Even after employing sophisticated multivariate machine learning methods that combined weak classifiers to enhance inference power, only the optimized CART model was able to improve predictive power over the univariate analyses alone. That model provided a specificity of 0.433, a sensitivity of 0.769, and an AROC of 0.601; which only modestly improves upon the sensitivity of univariate models but improves significantly upon the sensitivity of a clinical prediction alone, where the sensitivity of toxicity prediction amounts to a mere 20%.

Influential task groups have established the need for innovative methods to inform normal tissue dose limits for SBRT while cautioning against direct extrapolation from conventional radiotherapy data (7). However, there is a paucity of data for guiding clinicians in this new space, other than binary dose/volume criteria routinely employed on prospective trials. Other groups have suggested there may be more complexity involved in predicting toxicity after SBRT (10), spurring the creation of “complementary critical volume constraints” (specifying a volume of parallel tissue that is allowed to receive a pre-specified threshold dose or less), which have routinely been integrated in SBRT trials within the NRG (11). This in turn prompted the hypothesis that the entire DVH might contain a wealth of useful information informing toxicity that may have previously gone underutilized.

An important finding of this study is that information taken from the entire bladder, rectal, and prostate DVHs, in conjunction with ADT use, can improve toxicity prediction over clinical models alone, but even advanced multivariate machine learning methods encountered a ceiling in terms of their ability to predict toxicity. This likely has several explanations. First, since all patients were required to meet institutional constraints shown in Table 1 prior to plan approval, DVH features were already uniform among our cohort of patients, making the identification of predictors within small deviations among a relatively homogenous subset of DVH features challenging and susceptible to noise. It is entirely possible that a similar analysis in a cohort of patients with more heterogeneous planning metrics may have led to a disparate conclusion. An alternative, non-mutually exclusive explanation is that dosimetric features alone are not the primary drivers of toxicity beyond a certain threshold. This, in turn, suggests that a patient's biological features, including genomic signatures known to regulate radiation response in normal tissues (12, 13), may play an important role in predicting toxicity after radiotherapy. While monogenic associations between germline mutations in key genes such as *ATM* have been associated with severe toxicity (14), such mutations are rare and are unlikely to explain observed rates of grade 2 or higher toxicity which approach 20%. A recently published genome-wide single nucleotide polymorphism study was able to identify a predictive model for a weak urinary stream in a similarly sized cohort of men treated with brachytherapy with or without external beam radiotherapy, but the AROC was still limited at 0.70 (15). It is possible that utilizing both dosimetric and biological variables may allow the construction of a highly predictive model. Alternatively, the ability to create such a model may require the addition of other genetic and dosimetric variables, not currently captured in the platforms used in these studies.

Notably, 8 of the top 10 candidate dosimetric features for predicting grade ≥2 late GU toxicity (ranked by AROC) were all high dose rectal parameters, and the other two were low dose rectal parameters. This likely reflects the collinearity of individual dosimetric parameters. However, it is also possible that, by minimizing hot spots in the rectum, there will be a commensurate increase in dose to bladder or urethral subvolumes. While higher doses in bladder subvolumes should have been captured in the present analyses, the urethra is not universally contoured in our workflow, and urethral overdosing might not have been detected. Additionally, certain bladder regions may be more susceptible than others, and these tradeoffs might not have been captured in our analysis.

Limitations of our study include its single-institution nature, which invites the potential for selection bias. Our outcomes are also physician-reported rather than patient-reported, which could have precluded patient entry into our model due to underestimation (16) of actual grade 2 toxicity. Our dosimetric study also did not capture all potential predictors of increased toxicity, and patient-specific variables such as baseline International Prostate Symptom (IPSS) score (17), patient age (18), or history of trans-urethral resection of the prostate, which have all been thought to increase the likelihood of toxicity following SBRT, were not considered in this study due to limitations in our data set. However, the true importance of these issues remains an open question, as a recent propensity score-matched study demonstrated no increase in the rate of acute and late GU toxicity in patients who had undergone prior TURP, for example (19). Importantly, size of the prostate gland, which has been implicated in increasing toxicity at the arbitrary cutoff of 50cc in some series (17) but not in others (20), was examined, and did not emerge as an important component in predicting late GU toxicity at volumes >50cc. We were unable to evaluate dosimetry for structures not routinely contoured at our institution, such as the urethra and the rectal wall (as opposed to the total rectum structure). As all of our plans are homogeneous and intra-prostatic “hot spots” are small in volume, we did not evaluate structures such as the volume of the prostate receiving >40 Gy. Strengths of our approach include our ability to sample information from the entire DVH rather than arbitrary cut points as well as the novelty of applying advanced methodologies rooted in machine learning. In conventional multivariate analyses, variables of interest are designated *a priori*, whereas more complex modeling allowed us to control potential confounding factors that could not be identified prospectively. While our sample size was relatively small for “big data” modeling strategies, oversampling was not a concern in our analysis, given the negative findings.

## Conclusions

New technologies for increasing tumor control, such as SBRT, must be accompanied by similarly innovative approaches for understanding and mitigating toxicity, especially in the immature space of normal tissue dose limits during hypofractionation. We confirm through multiple iterative machine-learned models that there is a ceiling beyond which data from the entire DVH cannot predict late GU toxicity. We postulate that a more formal understanding of biological, rather than dosimetric features will allow us to maximize the therapeutic ratio by predicting and mitigating toxicity associated with SBRT.

## Data Availability Statement

The datasets generated for this study are available on request to the corresponding author.

## Ethics Statement

The studies involving human participants were reviewed and approved by the Medical Institutional Review Board 2 (MIRB2) at UCLA's Office of the Human Research Protection Program. The patients/participants provided their written informed consent to participate in this study.

## Author Contributions

LV, AK, and DR conceived and designed the analysis. AD, AP, NN, DL, XQ, CK, AK, and MC collected the data. JW, PL, MS, PK, CK, and AK contributed data or analysis tools. DR performed the analysis. LV, DR, AD, RL-E, PL, DL, and AK wrote the manuscript, and MS, PK, and CK provided mentorship and guidance.

## Conflict of Interest

The authors declare that the research was conducted in the absence of any commercial or financial relationships that could be construed as a potential conflict of interest. The handling Editor declared a past co-authorship with one of the authors AK.

## Supplementary Material

Supplemental Figure 1 Optimized dosimetric prediction tree with the number of patients at risk for developing late grade 2 or greater GU toxicity represented at each rectangular node. Tree has been trimmed to illustrate the primary branches of significance and dosimetric cut-points are underlined. Patients with and without toxicity according to the preceding cut-point are indicated in red and blue, respectively. The AROC corresponding to this optimized tree can be found in Figure 1B.
