# Supplementary figures and images for: Development and Validation of a Comprehensive Multivariate Dosimetric Model for Predicting Late Genitourinary Toxicity Following Prostate Cancer Stereotactic Body Radiotherapy
Source: Front Oncol. 2020 May 20;10:786. doi: 10.3389/fonc.2020.00786 (PMC7251156; doi:10.3389/fonc.2020.00786)

**Supplemental Figure 1: Optimal Classification and Regression Tree**


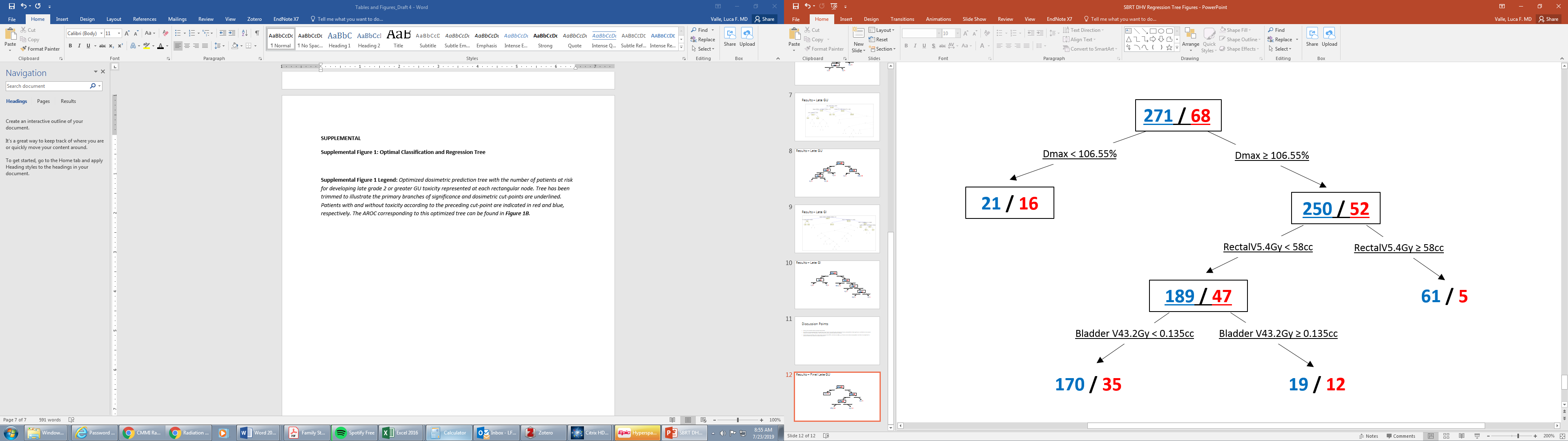

Supplement: Supplemental Figure 1 — Optimized dosimetric prediction tree with the number of patients at risk for developing late grade 2 or greater GU toxicity represented at each rectangular node. Tree has been trimmed to illustrate the primary branches of significance and dosimetric cut-points are underlined. Patients with and without toxicity according to the preceding cut-point are indicated in red and blue, respectively. The AROC corresponding to this optimized tree can be found in Figure 1B. [file Data_Sheet_1.DOCX]
